# Association between self-reported vision and mental well-being: a cross-sectional secondary analysis of Health Survey for England data

**DOI:** 10.1136/bmjopen-2025-101753

**Published:** 2025-08-16

**Authors:** Michael D Crossland, Tessa M Dekker, Marc S Tibber

**Affiliations:** 1 UCL Institute of Ophthalmology, London, UK; 2 NIHR Moorfields Biomedical Research Centre, London, UK; 3 University College London Division of Psychology and Language Sciences, London, UK; 4 Department of Clinical, Educational and Health Psychology, University College London, London, UK

**Keywords:** Health, OPHTHALMOLOGY, MENTAL HEALTH

## Abstract

**Objectives:**

Eye disease and vision impairment are known to be associated with reduced mental well-being, but less is known about the well-being of people with near-normal levels of vision. Here, we examined the association between self-reported eyesight and mental well-being, controlling for eye disease, mental ill-health and demographic factors, for adults with a wide range of age and vision.

**Design:**

Population-based cross-sectional study.

**Participants:**

7705 adults (56% women; median age 49 years, range 16–104 years) who participated in the Health Survey for England 2013, self-reported their eyesight status and completed the Warwick-Edinburgh Mental Well-Being Scale.

**Primary outcome measure:**

Mental well-being, controlling for self-reported mental ill health, self-reported eye disease, age, sex, socioeconomic group and ethnic origin.

**Results:**

Poorer self-reported eyesight was strongly associated with lower mental well-being (univariate linear model, F_(4,7700)_=94.7, p<0.001) and explained 5% of the variance in the outcome variable (R^2^=0.047). Relative to reporting ‘poor’ vision, each subsequent level of vision predicted better well-being, with the exception of ‘fair’ vision, which was not significantly different from ‘poor’ reported vision. This association remained significant after controlling for self-reported mental ill health, self-reported eye disease, age, sex, socioeconomic group and ethnic origin.

**Conclusions:**

Self-reported eyesight is strongly associated with mental well-being, irrespective of whether people have vision impairment or a diagnosed eye disease. This relationship exists in people with and without mental ill-health. Mental well-being should be considered in people with reduced eyesight, regardless of whether they have a diagnosed eye disease or mental ill-health. Interventions which improve vision may have a positive impact on mental well-being.

**Strengths and limitations of this study**

- The study has a large sample size and uses a robust measure of mental well-being.
- Multiple potentially confounding factors that may affect well-being have been controlled for, including mental ill-health, demographic and socioeconomic characteristics.
- We rely on self-reports of eyesight, eye disease and mental ill-health rather than more objective measures.
- The cross-sectional nature of the Health Survey for England means we are unable to determine causality between self-reported vision and well-being.

## Introduction

 Almost 2 million people in the UK have vision impairment,^1^ of whom about 134 000 are registered as sight impaired (formerly called ‘partially sighted’) and around the same number are registered severely sight impaired (previously ‘blind’).^2^ People with vision impairment experience poorer mental well-being,^3^ lower life satisfaction^4^ and increased psychological distress^5^ relative to those without. In a longitudinal study of older adults, Xiang and colleagues found that vision had a stronger association than dementia on well-being, but that depression had an even greater role on well-being.^3^

Many people have reduced eyesight yet do not meet the WHO’s definition of vision impairment (broadly speaking, those with visual acuity less than 6/12 in their better eye, or with significant visual field loss). This can be due to mild eye disease such as early cataract, uncorrected refractive error, amblyopia (often caused by suboptimal eye care in early life) or not having access to the optimal spectacle correction. In a large UK-based population study, only 77% of adults were found to have ‘normal’ vision in both eyes with their current spectacles or contact lenses (if worn).^6^

The impact of this subthreshold vision impairment on well-being is not well understood, despite the implications this might have for treatment and intervention. For example, if early vision impairment was associated with poorer well-being, this would encourage earlier treatment for diseases like cataract. One study has shown a relationship between poorer self-reported vision, tearfulness, lack of enjoyment, hopelessness, difficulty concentrating and other aspects of well-being.^7^ However, this study only investigated older adults, did not use a standardised measure of mental well-being and did not correct for the higher levels of depression also found in people with poorer levels of eyesight.

The interaction between self-reported vision and well-being is complicated by the well-established links between eye disease, depression^8^ and anxiety.^9^ For example, the odds of experiencing depression are approximately doubled in people with vision impairment.^10^ However, this is potentially confounded by activity limitation (a common ‘symptom’ of depression), which can be incorrectly attributed to depression, yet actually caused by vision impairment: somebody may respond ‘yes’ to the screening question ‘have you dropped many of your activities and interests?’ not because they are depressed, but because they are no longer able to see well enough to continue their hobbies.^11^ It is unclear whether the lower well-being experienced by people with vision impairment is simply caused by the increased level of depression and mental ill health in this population, or whether vision impairment has an independent impact on well-being.

The interaction between vision impairment and well-being is further complicated by demographic, health and societal factors, many of which are associated with an increased likelihood of vision impairment and eye disease, as well as mental ill-health and poor well-being.^612^,^17^ For example, experience of childhood poverty has been linked to increased rates of a range of mental health difficulties, including depression,^18 19^ as well as physical health difficulties, including low vision.^14 20^

In this study, we performed a secondary analysis on a large dataset of adults with a wide age range to examine the relationship between eyesight and mental well-being while controlling for mental ill-health, demographic and socioeconomic characteristics. This is an extremely rich dataset which allows for multiple evaluations of factors which may affect well-being, but to reduce the risk of a type I error (false positive findings), we limited our analysis to self-reported vision, eye disease, mental ill-health and demographic items. These factors were determined by our clinical experience and knowledge gaps in the existing research literature. Our primary hypothesis was that reduced vision would be associated with poorer well-being. Our secondary hypotheses were that this effect would survive after controlling for (1) mental ill-health, (2) eye disease and (3) demographic and socioeconomic factors. Confirmation of these secondary hypotheses would have potential implications for the ophthalmological treatment of people with early eye disease, and for the provision of interventions to improve well-being in people reporting reduced vision.

## Method

A secondary analysis of data collected as part of the 2013 Health Survey for England (HSE) was undertaken. HSE includes a large number of participants, representative of the adult population of England, which has been administered annually since 1991^21^ and since 2010 has included a self-reported assessment of mental well-being. In 2013, HSE additionally included items on vision, including self-reported eyesight when wearing spectacles or contact lenses and the presence of diagnosed eye disease. These data are available from the UK data service.^22^

### Participant identification

For the original study, participants were identified using a random selection of 9408 addresses in 588 postcode sectors across England. Every adult and up to two of the children in each selected household were eligible to participate. Data were collected in two stages. In the first, each participant completed an interview undertaken by a researcher and was given a self-completion booklet. The second involved a nurse visit, during which additional questions were asked and a blood sample was taken.

### Demographic details

Age at last birthday and ethnic group were self-reported. Household socioeconomic group was coded by the original study team by deriving the National Statistics Socio-economic Classification (NS-SEC)^23^ from the occupation of a ‘reference person’ within the household. The reference person was defined as the person in whose name the property was owned or rented; if there was more than one, the person with the highest income was used. If there were two householders with equal income, then the oldest person was chosen as the household reference person.^21^ For our analyses, the NS-SEC3 three-category scale was used, where socioeconomic status is classified as: (1) higher managerial/professional occupations, (2) intermediate occupations, (3) routine and manual occupations, with an option to report and (4) other.

### Level of vision and eye disease

In the first stage of data collection for the 2013 HSE, participants were asked, ‘Using glasses or corrective lenses if you use them, is your eyesight excellent, very good, good, fair or poor?’. We refer to these ordinal data as ‘self-reported vision’ throughout this paper.

Participants were also asked whether they had any ‘health conditions, illnesses or impairments, lasting or expected to last 12 months or more’ and were able to list up to six conditions. These conditions were coded by the interviewer. Participants were identified as having eye disease when the interviewer coded ‘cataract/poor eye sight/blindness’ or ‘other eye complaints’.

Additionally, participants were asked whether a doctor or optician had ever told them they had macular degeneration, cataract (and, if so, whether they had had surgery), diabetic eye disease or diabetic retinopathy, glaucoma or suspected glaucoma, injury or trauma resulting in loss of vision, or another serious eye condition; and if they were registered as ‘blind or partially sighted’ due to age-related macular degeneration, cataract, diabetic retinopathy, glaucoma, stroke or other neurological condition, or another cause. A positive response to any of these questions led to the participant being coded as having an eye disease (binary coding variable: presence/absence), except where the only disease reported was cataract, they reported having had surgery and they were not registered as sight impaired.

### Mental ill-health

Mental ill-health was defined when the code ‘mental illness/depression/anxiety/nerves’ was used at least once by the interviewer in response to the question about long-term health conditions(binary coding variable: presence/absence).

### Mental well-being

At the second stage of data collection, participants over 16 years of age were asked to self-complete the Warwick-Edinburgh Mental Well-Being Scale (WEMWBS),^24^ a 14-item questionnaire to assess positive affect, satisfaction with interpersonal relationships and positive functioning. This instrument has been shown to be unidimensional and to have strong internal consistency, construct validity and reliability.^25^

### Statistical analyses

To test our hypotheses, a series of linear regression analyses was undertaken. First, a correlation matrix was constructed to show correlations between the variables. To test the association between self-reported vision and well-being, well-being was regressed on self-reported vision. Well-being was a continuous variable (total WEMWBS score) and self-reported vision was an ordinal variable at five levels (excellent, very good, good, fair and poor). Given the extremely large age range of our participants, this analysis was repeated when stratified by age group (adolescents and emerging adults (age 16–29 years), adults (30–64 years) and older adults (≥65 years)). Having tested the primary association, further analyses determined whether this effect persisted after controlling for key covariates. Specifically, to test whether this association persisted when controlling for mental ill-health, the model was re-run with mental ill-health included as a covariate (categorical variable at two levels, multivariate model 1). Next, the model was re-run with eye disease included (categorical variable at two levels) to control for the effect of eye disease (multivariate model 2). Finally, the model was re-run with mental ill-health, eye disease, age, sex, socioeconomic status and ethnic group (white British or other) added simultaneously (multivariate model 3). The method of least squares was used to construct exact F-tests to determine the difference in well-being associated with each level of self-reported vision for each model. A Bonferroni correction was applied to adjust final p values to account for multiple comparisons made during model construction. All analyses were performed in JMP (Pro V.18.0.2, JMP Statistical Discovery, Cary, North Carolina, USA).

### Patient and public involvement

This analysis was motivated by the author MDC’s clinical observations of reduced well-being in people with mild vision impairment. The importance of the research was determined by discussion with representatives from patient groups (primarily Stargardt’s Connected and the Macular Society). Dissemination to patient communities has been performed at the Moorfields Biomedical Research Centre ‘Coffee Hour’ by author MDC and is planned for further patient groups.

### Publication of protocol, trial registration and preprint information

The method for HSE has been published previously,^26^ but the protocol for this secondary analysis was not pre-published. Neither HSE nor this secondary analysis was pre-registered on a trials database. A preprint version of this manuscript has been published.^27^

## Findings

**Figure 1:**
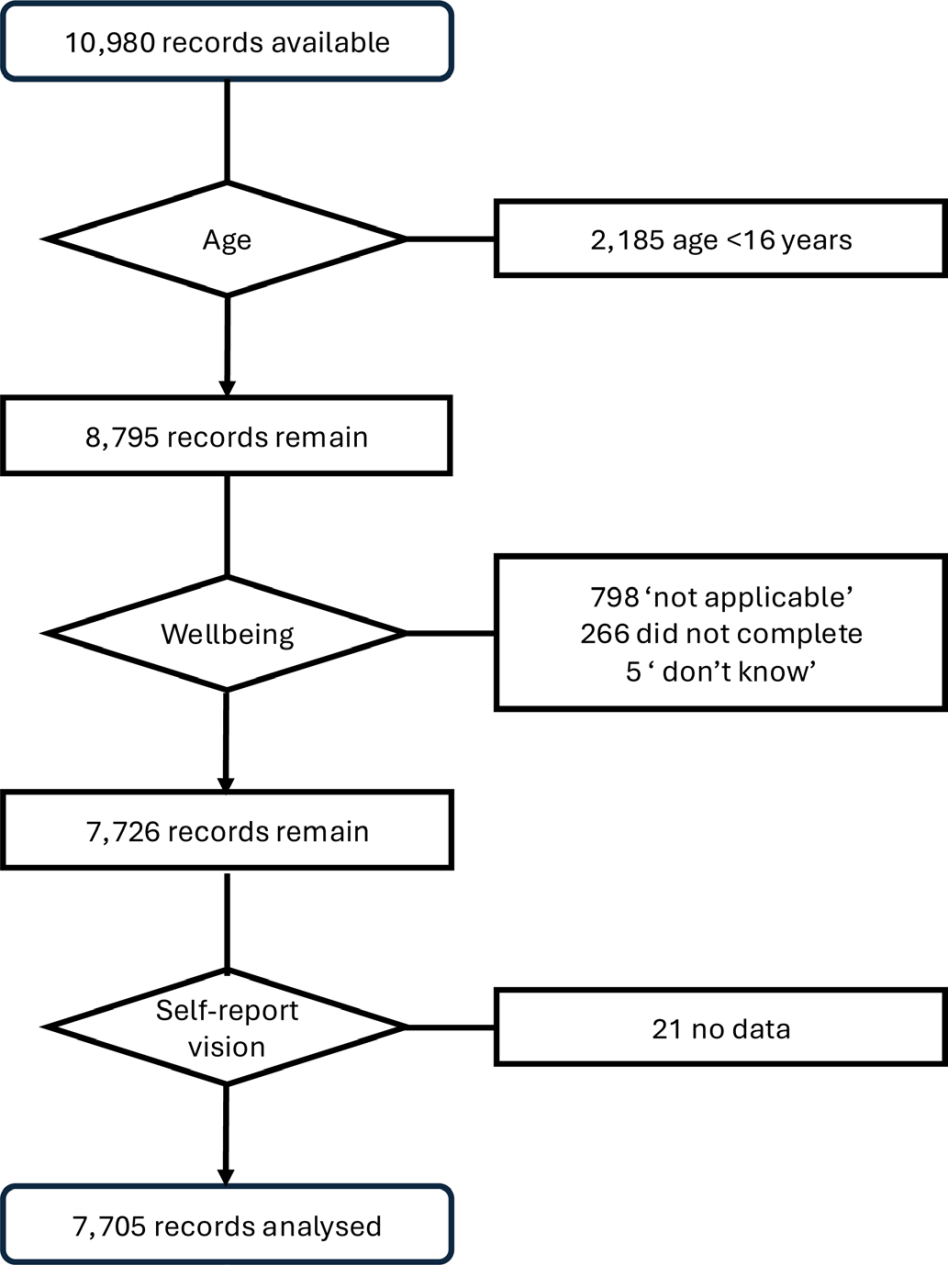
Flowchart showing how final analytical sample was reached.

Data were available for 10 980 participants, of whom 8795 were over 16 years of age. Mental well-being data were not available for 1069 adult participants: 266 participants chose not to complete this questionnaire, five answered ‘don’t know’, and for 798 data were scored as ‘not applicable’, largely due to the nurse visit not being completed. Well-being data were available for 81.3% of those with ‘fair’ or ‘poor’ vision, compared with 88.6% of those reporting better vision, a statistically significant difference (Pearson χ^2^=35.7, p<0.0001). Self-reported vision status was not available for a further 21 people. Data from the remaining 7705 participants were used for subsequent complete case analyses (figure 1). This final sample included 3391 men and 4313 women, with mean age 49.5 years (SD: 18.3; range 16–104). Characteristics of the study population are shown in table 1.

**Table 1:** Characteristics of study population stratified by level of vision

	Excellent N=2383	Very good N=2449	Good N=2249	Fair N=509	Poor N=115	Variance between groups
Age (years), mean (SD)	42.5 (17)	49.9 (18)	54.3 (17)	56.6 (18)	56.3 (21)	F_(4,7700)_=160 p<0.001
Sex, number (%) female	1236 (51%)	1405 (57%)	1314 (58%)	279 (55%)	80 (70%)	χ^2^=232.6 p<0.001
Ethnic origin, number (%)						
White British	1952 (82%)	2066 (84%)	1927 (86%)	447 (88%)	97 (84%)	χ^2^*=*287.9 p=0.10
White Irish	17 (0.7%)	10 (0.4%)	21 (1%)	2 (0.4%)	2 (2%)	
White Traveller	2 (0.001%)	0	1 (0.04%)	0	0	
Any other white background	126 (5.3%)	121 (5%)	78 (3%)	20 (4%)	1 (1%)	
White and Black Caribbean	16 (0.7%)	9 (0.4%)	7 (0.3%)	3 (1%)	1 (1%)	
White and Black African	6 (0.3%)	5 (0.2%)	1 (0.04%)	1 (0.2%)	0	
White and Asian	8 (0.3%)	4 (0.2%)	6 (0.3%)	0	0	
Any other mixed/multiple	10 (0.4%)	11 (0.4%)	9 (0.4%)	2 (0.4%)	1 (1%)	
Indian	63 (3%)	63 (2.6%)	48 (2%)	9 (2%)	4 (3%)	
Pakistani	33 (1%)	22 (0.9%)	17 (0.8%)	5 (1%)	3 (3%)	
Bangladeshi	14 (0.6%)	16 (0.1%)	13 (0.6%)	1 (0.2%)	0	
Chinese	8 (0.3%)	10 (0.4%)	13 (0.6%)	3 (1%)	1 (1%)	
Any other Asian background	32 (1.3%)	38 (1.6%)	38 (1.7%)	3 (1%)	0	
African	47 (2%)	27 (1%)	24 (1%)	6 (1%)	1 (1%)	
Caribbean	19 (0.8%)	19 (0.8%)	19 (1%)	4 (1%)	1 (1%)	
Any other Black/African/Caribbean background	9 (0.4%)	2 (0.1%)	3 (0.1%)	2 (0.4%)	1 (1%)	
Arab	4 (0.2%)	7 (0.3%)	4 (0.2%)	0	0	
Any other ethnic group	15 (0.6%)	18 (0.7%)	17 (0.1%)	1 (0.2%)	0	
No answer/refused	2 (0.1%)	1 (0.04%)	3 (0.1%)	0	0	
Socioeconomic status						
Managerial and professional occupations, number (%)	1101 (46%)	1041 (43%)	834 (37%)	157 (31%)	27 (23%)	χ^2^*=*2117 p<0.001
Intermediate occupations, number (%)	525 (22%)	547 (22%)	508 (23%)	107 (21%)	22 (19%)	
Routine and manual occupation, number (%)	682 (29%)	806 (33%)	851 (38%)	225 (44%)	59 (51%)	
Other, number (%)	73 (3%)	52 (2%)	56 (2%)	20 (4%)	7 (6%)	
Mental health condition (yes), number (%)	122 (5%)	118 (5%)	132 (6%)	56 (11%)	14 (12%)	χ^2^=240.5 p<0.001
Eye condition (yes), number (%)	171 (7%)	311 (13%)	478 (21%)	174 (34%)	64 (56%)	χ^2^=2474 p<0.001
Well-being, mean (SD)	53.3 (8)	51.8 (8)	50.3 (9)	46.7 (9)	46.6 (11)	F_(4,7700)_=94.7 p<0.001

Correlations between the variables are shown in the matrix in table 2. In summary, older participants were more likely to be female, have lower socioeconomic status, be white British, report a mental health condition and report an eye condition. Female participants were more likely to have lower socioeconomic status and mental ill-health. People from lower socioeconomic groups were more likely to be non-white British, to have mental ill-health, to have an eye condition and to have lower well-being. Participants who were not white British were more likely to have a mental health condition or an eye condition, but had higher mental well-being. People with mental health conditions had lower well-being, as did those with eye conditions.

**Table 2:** Correlation matrix showing relationship between variables

	Age	Sex	SES	Origin	MH condition	Eye condition
Sex	**χ^2^=8.71** **p<0.01**	–				
SES	**χ^2^=6.48** **p<0.05**	**χ^2^=21.0** **p<0.01**	–			
Origin	**χ^2^=359** **p<0.001**	χ^2^=20.44 p=0.51	**χ^2^=148** **p<0.001**	–		
MH condition	**χ^2^=25.3** **p<0.001**	**χ^2^=8.71** **p<0.01**	**χ^2^=38.3** **p<0.001**	**χ^2^=15.4** **p<0.001**	–	
Eye condition	**χ^2^=1460** **p<0.001**	χ^2^=21.19 p=0.27	**χ^2^=19.1** **p<0.001**	**χ^2^=62.7** **p<0.001**	χ^2^=23.44 p=0.06	–
Well-being	R^2^=0.00 p=0.95	t=−1.52 p=0.94	**F=58.3** **p<0.001**	**t=−4.83** **p<0.001**	**t=−26.8** **p<0.001**	**t=−4.12** **p<0.001**

The tests reported are: Pearson χ2 (for correlations between nominal variables and nominal variables), Student’s t-test (for correlations between nominal data and continuous variables), linear regression (for correlations between continuous variables and continuous variables).

Significant correlations at p<0.05 are shown in bold.

MH condition, mental health condition; SES, socioeconomic status.

**Figure 2:**
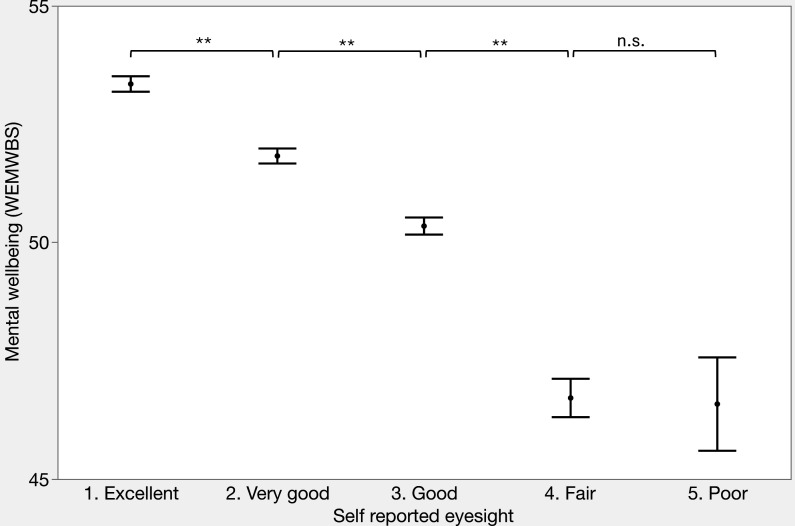
The relationship between self-reported vision and mental well-being. Points represent mean values. Error bars show SE of the mean. Dashed line separates those defined as having vision impairment from those without. **Significant at p<0.0001 (Student’s t*-*test). n.s., not significant, p>0.05. WEMWBS, Warwick-Edinburgh Mental Well-Being Scale.

Self-reported vision was associated with poorer mental well-being (exact F-test, using method of least squares: F_(4,7700)_=94.7, p<0.001, figure 2, table 1) and explained 5% of the variance in the outcome variable (R^2^=0.047). Relative to reporting ‘poor’ vision, each subsequent level of vision predicted better well-being, with the exception of ‘fair’ vision, which was not significantly different from ‘poor’ vision (table 3). This effect persisted across all age categories (online supplemental table 1), although there was no difference in well-being between ‘good,’ ‘fair’ and ‘poor’ vision for the youngest participants.

**Table 3:** Regression analyses showing the regression of well-being on self-reported vision, mental ill-health, eye disease, demographic and socioeconomic variables

Predictor	Level	Univariate models	Multivariate model 1	Multivariate model 2	Multivariate model 3
		Coefficient (95% CI)	Coefficient (95% CI)	Coefficient (95% CI)	Coefficient (95% CI)
Self-reported vision	Excellent	**6.05** **(4.57 to 7.53) p<0.0001**	**6.05** **(4.57 to 7.53) p<0.001**	**6.81** **(5.25 to 8.38) p<0.001**	**5.66** **(4.17 to 7.16) p<0.001**
	Very good	**4.49** **(3.01 to 5.98) p<0.0001**	**4.49** **(3.01 to 5.98) p<0.001**	**5.29** **(3.73 to 6.85) p<0.001**	**4.04** **(2.55 to 5.53) p<0.001**
	Good	**3.12** **(1.64 to 4.60) p<0.0001**	**3.12** **(1.64 to 4.60) p<0.001**	**3.80** **(2.24 to 5.35) p<0.001**	**2.71** **(1.23 to 4.19) p<0.01**
	Fair	0.01 (−1.59 to 1.61) p=0.99	0.01 (−1.59 to 1.61) p=1.0	0.15 (−1.52 to 1.83) p=1.0	−0.24 (−1.83 to 1.35) p=0.77
Mental ill-health	No	**10.6** **(9.82 to 11.4) p<0.0001**	**10.2** **(9.40 to 10.9) p<0.001**	–	**9.73** **(8.97 to 10.5) p<0.001**
Eye disease	No	**1.11** **(0.59 to 1.63) p<0.0001**	–	0.10 (−0.42 to 0.62) p=0.70	0.61 (0.07 to 1.15) p=0.19
Sex	Female	−0.30 (−0.68 to 0.08) p=0.13	–	–	0.08 (−0.43 to 0.28) p=0.67
Age		0.00003 (−0.01 to 0.01) p=0.95	–	–	**0.03** **(0.02 to 0.04) p<0.001**
Ethnicity	Other	**1.28** **(0.76 to 1.80) p<0.0001**	–	–	**1.14** **(0.64 to 1.64) p<0.001**
Socioeconomic status	Higher managerial or professional	**2.84** **(2.41 to 3.27) p<0.0001**	–	–	**2.13** **(1.72 to 2.54) p<0.001**
	Intermediate	**1.07** **(0.56 to 1.58) p<0.0001**	–	–	**0.67** **(0.20 to 1.15) p<0.05**
	Other	0.097 (−1.08 to 1.28) p=0.87	–	–	0.07 (−1.05 to 1.19) p=1.0

Values in bold indicate significant predictors at p<0.05. P values for multivariate models are adjusted using the Bonferroni correction. Reference levels for categorical predictors were (in brackets) as follows: self-reported vision (poor), mental ill-health (yes), eye disease (yes), gender (male), ethnicity (white British), socioeconomic status (routine or manual).

This association remained significant when mental ill-health (multivariate model 1, exact F-test, using method of least squares, F_(5,7699)_=87.3, p<0.001, R^2^=0.12) and eye disease (multivariate model 2, exact F-test, using method of least squares, F_(5,7699)_=90.2, p<0.001, R^2^=0.047) were added (separately) as covariates. Further, the relationship remained significant after sex, age, ethnic group and socioeconomic group were added (simultaneously) as further covariates alongside mental ill-health and eye disease (multivariate model 3, exact F-test, using method of least squares, F_(29,7670)_=76.9, p<0.001, R^2^=0.14).

## Discussion

In support of our primary hypothesis, that reduced vision is associated with poorer well-being, we have shown a robust association between self-reported vision and mental well-being. Further, consistent with our secondary hypotheses that this effect would survive after controlling for mental ill-health, eye disease and demographic and socioeconomic factors, this relationship remained significant. Our analyses did not show a difference in well-being between those with ‘fair’ vision and those reporting ‘poor’ vision. Self-reported ‘fair’ or ‘poor’ vision has been shown to be sensitive and specific for defining vision impairment in analyses of data from older adults in three studies (the Irish Longitudinal Study on Ageing, the MRC Assessment and Management of Older People in the Community trial and the Health and Retirement Survey).^28 29^ This suggests a plateau effect in our data: progressively poorer vision correlates with poorer well-being until the level of ‘vision impairment’ is reached, after which changes in vision are not associated with further lowering of well-being scores.

Strengths of this study include its large, representative and wide-ranging population of participants, its use of a reliable and well-validated instrument to assess well-being, and our inclusion of multiple potential confounders in our statistical modelling. There are four key weaknesses. First, we used self-reported measures of vision and mental health status. Although there is a precedent for using self-reported vision status in population studies,^3 5 30^ this may lead to a small bias towards over-reporting^28 29^ or under-reporting^31^ of vision impairment. We also relied on self-reports of mental ill-health and eye disease, the results of which may have been limited by recall bias, by not reporting undiagnosed mental health conditions, or by self-diagnosis of mental health conditions without diagnostic criteria being met. Second, the cross-sectional nature of the study precludes our ability to determine causality or the direction of causality. For example, it is possible that people with reduced well-being tend to underestimate how good their vision is, although depression itself is not generally associated with inaccurate visual acuity testing.^32^ Although HSE has been performed for many years, the same participants are not included in every survey—a new sample is recruited each year. This allows for trend analyses, but not for longitudinal assessments of change within an individual. A previous longitudinal study has shown a bidirectional relationship between anxiety and vision impairment, where people with vision impairment experienced greater anxiety, but people with anxiety were also more likely to become vision impaired, perhaps due to late presentation to services, poor lifestyle factors (such as diet) or their anxiety being caused by having a vision-threatening condition.^27 28^ A third possible limitation relates to data collection: the WEMWBS was self-completed by participants as part of a booklet, which is likely to have made it less accessible to people with vision impairment, reflected in the fact that well-being data were available for fewer people reporting ‘fair’ or ‘poor’ vision. Finally, the data we are analysing are now twelve years old and it is possible that the relationship between vision and well-being may have changed over the past few years, perhaps due to an increase in loneliness following the COVID-19 pandemic,^33 34^ or the increase in digital inclusion over this time period.^35^ However, the 2013 survey was the most recent to include questions on eyesight, as this is one of the ‘special topics’ which are only included in 1 year’s version of the survey.

Our findings build on existing research into well-being in people with vision impairment^3 4^ and show that even among those without vision impairment, better self-reported vision is associated with better mental well-being. This finding agrees with Mojon-Azzi and colleagues,^7^ who also found an association between self-reported vision and certain aspects of well-being in an older adult sample. Our study supports and extends these findings to a much wider age range and uses a validated and more rigorous instrument to assess well-being (the WEMWBS). Our results also reflect the findings of Cumberland *et al*, who used UK Biobank data to show that mildly reduced vision was associated with increased mental ill-health and poorer self-rated general health.^6^ This work further extends previous research by showing that the effects persist after controlling for a number of potential key confounders. For example, the association between self-reported vision and well-being applied to those with and without a diagnosis of mental ill-health. This suggests that, consistent with stepped care and early intervention models of intervention, people with vision impairment or who report reduced vision may benefit from support to improve their well-being, even if they do not have a diagnosis of depression or any other mental health condition. Our findings are consistent with calls for the integration of mental and physical healthcare, improving access to mental health services by self-referral, and the provision of mental health support in physical health services.^36^

The poorer well-being we have identified in people with poorer vision may represent subclinical depression^37^ or may reflect people who have depression but have not been formally diagnosed. Nollett and colleagues have shown that many people with vision impairment display signs of depression but are not receiving treatment.^38^ Early mental health intervention has been suggested as an important component of vision rehabilitation,^39^ although the evidence base for psychosocial interventions improving the mental health of people with vision impairment remains limited.^40^ Incorporating mental health support within eye clinics may support positive mental health outcomes.^41 42^ The fact that well-being is reduced even in people who rate their vision as ‘very good’ or ‘good’ relative to ‘excellent’ suggests that well-being may be increased by early treatment of eye disease, for example, surgery for mild cataract, which might improve vision from ‘good’ to ‘very good’. People without eye disease also showed an association between self-reported vision and mental well-being. This may reflect undiagnosed eye disease, or the need for an updated refractive correction in people who need spectacles but do not have an eye disease.

Future research should adopt a longitudinal design to determine whether ophthalmological intervention in early eye disease does indeed improve well-being and whether psychological treatments, such as stepped care,^43^ intended to improve well-being, can prevent the subsequent development of depression in people with vision impairment. This would support calls for integrating mental and physical health services.^42 44^ Our data are not necessarily generalisable and it would be interesting to examine whether the same relationship between well-being and self-reported vision exists in other contexts, such as people in residential care or those living in other countries. The well-being of people from lower socioeconomic groups, who are known to have poorer access to eyecare services,^45^ also merits further research.

## Supplementary material

10.1136/bmjopen-2025-101753 online supplemental file 1

## Data Availability

Data are available in a public, open access repository.
