# Supplementary material for: Association between self-reported vision and mental well-being: a cross-sectional secondary analysis of Health Survey for England data
Source: BMJ Open. 2025 Aug 16;15(8):e101753. doi: 10.1136/bmjopen-2025-101753 (PMC12359461; doi:10.1136/bmjopen-2025-101753)
Supplement: online supplemental file 1 [file bmjopen-15-8-s001.docx]

**Supplementary table 1.** Regression analyses showing the regression of wellbeing on self-reported vision, mental ill-health, eye disease, demographic and socioeconomic variable, stratified by age category. Values in bold indicate significant predictors at p<0.05. Reference levels for categorical predictors were (in brackets) as follows: self-reported vision (poor), mental ill-health (yes), eye disease (yes), gender (male), ethnicity (white British), socioeconomic status (routine or manual).

|  | | **Adolescent and emerging adult**  **(16-29 years, n=1252)** | | **Adult**  **(30-64 years, n=4577)** | | **Older adult**  **(≥65 years,**  **n=1876)** | |
| --- | --- | --- | --- | --- | --- | --- | --- |
| **Predictor** | **Level** | Coefficient  (95% CI) | *p* value | Coefficient  (95% CI) | *p* value | Coefficient  (95% CI) | *p* value |
| Self-reported vision | Excellent | **1.67**  **(0.65, 2.69)** | **<0.0001** | **6.78**  **(4.52, 9.04)** | **<0.0001** | **8.91 (6.30, 11.5)** | **<0.0001** |
|  | Very good | **3.85**  **(0.09, 7.61)** | **<0.0001** | **5.34**  **(3.08, 7.60)** | **<0.0001** | **6.03**  **(3.50, 8.55)** | **<0.0001** |
|  | Good | 1.09  (-2.73, 4.92) | 0.57 | **3.94**  **(1.68, 6.21)** | **<0.0001** | **4.56**  **(2.05, 7.08)** | **<0.0001** |
|  | Fair | -1.57  (-5.93, 2.80) | 0.48 | -0.26  (-2.68, 2.16) | 0.83 | 1.42  (-1.29, 4.13) | 0.30 |
| Mental ill-health | No | **8.92 (7.27, 10.6)** | **<0.0001** | **11.5**  **(10.6, 12.5)** | **<0.0001** | **7.77 (5.32, 10.2)** | **<0.0001** |
| Eye disease | No | 2.09  (-0.40, 4.58) | 0.10 | **1.68 (0.76, 2.61)** | **<0.001** | **1.76**  **(1.00, 2.53)** | **<0.0001** |
| Sex | Female | 0.77  (-0.15, 1.68) | 0.10 | -0.04  (-0.54, 0.46) | 0.88 | 0.71  (-0.05, 1.48) | 0.07 |
| Age |  | 0.055  (-0.55, 0.55) | 0.31 | -0.02  (-0.046, 0.006) | 0.08 | **-0.156**  **(-0.21,**  **-0.1)** | **<0.0001** |
| Ethnicity | Other | **1.65**  **(0.57, 2.73)** | **<0.01** | **1.39**  **(0.74, 2.03)** | **<0.0001** | 1.11  (-0.50, 2.71) | 0.18 |
| Socioeconomic status | Higher managerial/  professional | **2.39**  **(1.33, 3.46)** | **<0.0001** | **3.44**  **(2.88, 4.00)** | **<0.0001** | **2.02**  **(1.13, 2.90)** | **<0.0001** |
|  | Intermediate | 0.74  (-0.48, 1.97) | 0.23 | **1.29**  **(0.61, 1.97)** | **<0.001** | 0.97  (-0.01, 1.95) | 0.05 |
|  | Other | -0.49  (-2.31, 1.32) | 0.59 | 0.69  (-1.15, 2.54) | 0.46 | -0.49  (-3.47, 2.48) | 0.75 |
