# Supplementary material for: Association between self-reported vision and mental well-being: a cross-sectional secondary analysis of Health Survey for England data
Source: BMJ Open. 2025 Aug 16;15(8):e101753. doi: 10.1136/bmjopen-2025-101753 (PMC12359461; doi:10.1136/bmjopen-2025-101753)
Supplement: Supplementary file 2 [file bmjopen-2025-101753.reviewer_comments.pdf]

PEER REVIEW HISTORY

BMJ Open publishes all reviews undertaken for accepted manuscripts. Reviewers are asked to complete a checklist review form and are provided with free text boxes to elaborate on their assessment. These free text comments are reproduced below.

ARTICLE DETAILS

Title (Provisional)

The association between self-reported vision and mental wellbeing: A cross-sectional secondary analysis of Health Survey for England data

Authors

Crossland, Michael D.; Dekker, Tessa M; Tibber, Marc Samuel

VERSION 1 - REVIEW

**Reviewer**	**1**
**Name**	**Dewi, Ayu Putu Madri**
**Affiliation**	**Amsterdam UMC Location AMC, Epidemiology and Data Science**
**Date**	**09-Apr-2025**
**COI**	**None**

Manuscript Title: The association between self-reported vision and mental wellbeing: A secondary analysis of Health Survey for England data

Dear authors,

I am an Open Science researcher, who has checked your manuscript for its adherence to a number of Open Science practices.

Below you find the checklist that I used to assess your manuscript for adherence to a number of these practices.

Based on this assessment, I would suggest the following refinements/additions:

1. Availability of a protocol: Please provide a (link to the) the protocol.

2. (Pre-)registration: Please provide information on whether and where the study was (pre-)registered.

3. Open Materials: OK

4. Open Data: Please provide a statement about whether and where the data used for this study can be accessed

5. Open Code: Please provide (a link to) the code used for the analyses in this study.

6. Citations: OK

7. Software and tools used are described: OK

8. Open access publication: OK

9. Reporting checklist provided: OK

10. Preprint: Please state explicitly whether and where the preprint of this study can be found.

Reviewer **2**

Name **Patnaik, Jennifer**

Affiliation **University of Colorado Denver - Anschutz Medical Campus, Ophthalmology**

Date **29-Apr-2025**

COI **None**

The research question is interesting and the study sample is large, but many items in the manuscript could be improved.

1. The abstract does not include a measure of association, which leaves the reader with very limited information.

2. I don't like the use of H1 and H2, it makes it very difficult to read because those of us less familiar with the study will not remember these full hypotheses. I would just write them out for clarity.

3. I think some additional statistical support is needed. It is not clear why self-reported vision was treated as an ordinal variable. Typically surveys like this are in no way ordinal. Multinomial would likely be a better option.

4. There is likely a lot of correlations between all of these variables, particularly with mental ill health, and wellbeing. There is absolutely no mention of correlation in this paper and it needs to be addressed.

5. The findings are so limited and presented in such a simple manner. There really should be a more thorough analysis of this rich data. After reading this paper, I felt very unsatisfied and like this research was quickly done without full thought or a full description of all that it has to offer.

6. The primary explanatory variable only explains 5% of the outcome. Do the full models do better in this regard? What are the main predictors of poor mental wellbeing.

Reviewer 3

Name OGLIARI, GIULIA

Affiliation Nottingham University Hospitals NHS Trust, Health Care for Older People (HCOP)

Date 02-May-2025

COI None

The paper by Crossland et al investigated the cross-sectional association between self-reported vision and mental wellbeing among 7,705 adults aged 16 years or older who participated in the Health Survey for England (HSE) 2013. My first comment is on the cross-sectional rather than longitudinal design. As the HSE has been administered annually for many years (and after 2013, please, correct me if I am wrong), the authors could have investigated the longitudinal association between self-reported eyesight at baseline (in 2013) and mental wellbeing at follow-up, in a population who did not have mental ill-health at baseline. My second comment is that the authors could have presented age-stratified analyses (for example, in younger adults, middle-aged adults and older adults, respectively), while still adjusting for age. I would suggest age-stratified analyses in view of the wide age range of their study population (16 to 104 years) and because the prevalence and risk factor for poorer wellbeing significantly vary across age categories.

I have a few other comments.

Title. Please, consider adding the word “cross-sectional” in the title. For example, “the cross-sectional association”.

Abstract. Please, consider reporting the age range and median age or mean age of the participants. Please, also consider reporting the proportion of women in your sample.

Strengths and weaknesses, page 4, lines 61-62. I would avoid the word “reduced” and change it with “poorer” or “lower”; in my view, the word “reduced” implies a change over time from a baseline. This study is only cross-sectional.

Methods, page 9. Please, mention whether the Health Survey for England may be representative of the community-dwelling population in England. Please, provide appropriate references.

Methods, page 10, lines 169-170. What happened in case the participants had more than six conditions?

Methods, page 12. Please, consider mentioning whether the Health Survey for England received Ethics approval from an institutional review board. Please, report the name of the institutional review board and the date of approval. I understand that no further Ethics approval was required for secondary data analyses, but you should report the original Ethics

approval. Please, also mention whether written informed consent was received from all participants. Please, mention whether and how capacity to consent to participation in this study was assessed and whether potential participants lacking capacity were excluded from this study.

Findings, page 13. Please, consider a flow chart to show how the final analytical sample was reached.

Findings. You should provide a “Table 1 Characteristics of study population”; you could consider presenting the characteristics in the five vision categories. In other words, the headings of your columns will be “excellent”, “very good”, “good”, “fair” and “poor” self-reported vision. In the rows, you will report the proportion of women, the mean age, the proportion of the ethnicities, those of socio-economic status. You may also test for differences in each of these characteristics across the categories of self-reported vision (for example, using chi-square test for categorical variables).

Findings, page 13. What kind of test is F, please? It may be unclear to the reader.

Discussion, page 17, lines 27-32. I am not sure whether the references correspond to these sentences. In the sentences, you mention three studies, but you only report two references, one of which refers to the English Longitudinal Study on Ageing rather than the Irish Longitudinal Study on Ageing.

Discussion, page 17, lines 8-19. Please, consider rephrasing this part by avoiding the use of the acronyms H1 and H2. For example, you could write “in a population of community-dwelling adults, we observed a cross-sectional association between poorer self-reported vision and poorer mental wellbeing”.

Discussion, page 18, lines 40-43. The statement “interventions to improve vision are likely to improve wellbeing” is not supported by your observational cross-sectional study. You may rephrase it (for example, “further research” or “further interventional studies should explore whether interventions to improve vision are likely to improve wellbeing”, or similar). You could also consider referencing previous interventional studies supporting this statement.

Discussion, page 18, lines 48-56. You should not present new results in the discussion. You should present them in the results section.

Discussion, page 19, line 56. Please, consider changing “reduced wellbeing” with “poorer wellbeing” for the reason I explained above.

Discussion, page 19 and reference 30. I would reference a study on the provision of mental health support in physical health services for adult patients, rather than paediatric patients, given that your study focused on adults aged 16 to 104 years.

VERSION 1 - AUTHOR RESPONSE

Reviewer: 1

Based on this assessment, I would suggest the following refinements/additions:

1. Availability of a protocol: Please provide a (link to the) the protocol.

The protocol for this analysis was not pre-published, but the protocol for the original data collection was. This has been added (section: 'Publication of protocol, trial registration and preprint information').

2. (Pre-)registration: Please provide information on whether and where the study was (pre-)registered.

Neither Health Survey for England nor this secondary analysis was pre-registered. This has been added (section: 'Publication of protocol, trial registration and preprint information').

3. Open Materials: OK

Thank you.

4. Open Data: Please provide a statement about whether and where the data used for this study can be accessed

This has been added.

5. Open Code: Please provide (a link to) the code used for the analyses in this study.

This is not applicable, analyses were performed in the graphical user interface of JMP. This has been added.

6. Citations: OK

Thank you.

7. Software and tools used are described: OK

Thank you.

8. Open access publication: OK

Thank you.

9. Reporting checklist provided: OK

Thank you.

10. Preprint: Please state explicitly whether and where the preprint of this study can be found.

This has been added.

Reviewer: 2

Comments to the Author:

The research question is interesting and the study sample is large, but many items in the manuscript could be improved.

1. The abstract does not include a measure of association, which leaves the reader with very limited information.

The results section of the abstract has been expanded, to read:

Poorer self-reported eyesight was strongly associated with lower mental wellbeing (univariate linear model, $F_{(4, 7700)} = 94.7$, $p < 0.001$) and explained 5% of the variance in the outcome variable (R2 = 0.047). Relative to reporting 'poor' vision, each subsequent level of vision predicted better wellbeing, with the exception of 'fair' vision, which was not significantly different to 'poor' reported vision. This association remained significant after controlling for self-reported mental ill health, self-reported eye disease, age, sex, socioeconomic group and ethnic origin.

2. I don't like the use of H1 and H2, it makes it very difficult to read because those of us less familiar with the study will not remember these full hypotheses. I would just write them out for clarity.
Changed as suggested – we agree this makes the manuscript more readable.

3. I think some additional statistical support is needed. It is not clear why self-reported vision was treated as an ordinal variable. Typically surveys like this are in no way ordinal. Multinomial would likely be a better option.
In response to this point, we wonder if there was some confusion as to what constituted the "self-reported vision" variable used in our analyses, likely driven by our failure to make this explicit. In the Methods, under the subsection titled 'Level of vision and eye disease' we reference a number of questions posed to the participants, including 'Using glasses or

corrective lenses if you use them, is your eyesight excellent, very good, good, fair or poor?' and asking them 'whether they had any 'health conditions, illnesses or impairments, lasting or expected to last 12 months or more'. To be clear, our self-reported vision variable comprised (only) participants' responses to the former question. Given that the response options were 'excellent,' 'very good,' 'good,' 'fair' or 'poor,' the scale of measurement is ordinal, since the response options can be easily ordered, though this of course is no guarantee that sequential response options relate to equal step sizes (hence why the variable does not meet the criteria for an interval scale of measurement). To clarify this, we now explicitly reference which item was used to create the 'self-reported vision' variable used in subsequent analyses.

To reassure the reviewer, we re-ran our models with multinomial regression and self-reported vision as a nominal variable. This did not alter the strength of the associations shown, or the validity of the models, giving remarkably similar results, confirming that ordinal classification was appropriate.

4. There is likely a lot of correlations between all of these variables, particularly with mental ill health, and wellbeing. There is absolutely no mention of correlation in this paper and it needs to be addressed.

Thank you for asking us to present these data, which we did analyse but did not present in the original paper. We have included a correlation matrix (table 2 and below), a description of this in the methods and

results. A Bonferroni correction was applied to adjust the final p values to account for the multiple comparisons made during model construction.

	Age	Sex	SES	Origin	MH condition	Eye condition
Sex	**χ^2 =8.71** **p < 0.01**	-				
SES	**χ^2 =6.48** **p < 0.05**	**χ^2 = 21.0** **p < 0.01**	-			
Origin	**χ^2 =359** **P < 0.001**	χ^2 = 0.44 p = 0.51	**χ^2 = 148** **p < 0.001**	-		
MH condition	**χ^2 =25.3** **p < 0.001**	**χ^2 = 8.71** **p < 0.01**	**χ^2 = 38.3** **p < 0.001**	**χ^2 = 15.4** **p < 0.001**	-	
Eye condition	**χ^2 = 1460** **p < 0.001**	χ^2 = 1.19 p = 0.27	**χ^2 = 19.1** **p < 0.001**	**χ^2 = 62.7** **p < 0.001**	χ^2 = 3.44 p = 0.06	-
Wellbeing	R^2 = 0.00 p = 0.95	t = -1.52 p = 0.94	**F = 58.3** **p < 0.001**	**t = -4.83** **p < 0.001**	**t = -26.8** **p < 0.001**	**t = -4.12** **p < 0.001**

5. The findings are so limited and presented in such a simple manner. There really should be a more thorough analysis of this rich data. After reading this paper, I felt very unsatisfied and like this research was quickly done without full thought or a full description of all that it has to offer.

We are pleased the reviewer agrees our research is presented in a simple manner, which was our intention, but saddened they were left unsatisfied.

We agree this is an extraordinarily rich dataset and there are multiple analyses which we could have performed. However, running multiple analyses without constraints risks inflating the likelihood of a type 1

error, i.e. false positives. Instead, we were specifically interested in the relationship between self-reported vision and mental wellbeing, and whether this is confounded by mental ill-health, guided by research, theory and practice and knowledge gaps, as outlined in the introduction. We have attempted to make our motivations clearer, by adding the following sentence:

> **This is an extremely rich dataset which allows for multiple evaluations of factors which may affect wellbeing, but to reduce the risk of a type I error (false positive findings) we limited our analysis to self-reported vision, eye disease, mental ill-health and demographic items. These factors were determined by our clinical experience and knowledge gaps in the existing research literature.**

6. The primary explanatory variable only explains 5% of the outcome. Do the full models do better in this regard? What are the main predictors of poor mental wellbeing.

The full models which include mental health condition do account for more of the variance in wellbeing: Multivariate model 1, r^2 = 0.12; Multivariate model 3, r^2 = 0.14; however, model 2 (adding eye disease) does not affect the model fit at all (r^2=0.047). This has been added to the results section. Given the complex and multiple factors which affect wellbeing, we feel that this relatively modest amount of variance accounted for is of clinical importance.

Reviewer: 3

The paper by Crossland et al investigated the cross-sectional association between self-reported vision and mental wellbeing among 7,705 adults aged 16 years or older who participated in the Health Survey for England (HSE) 2013. My first comment is on the cross-sectional rather than longitudinal design. As the HSE has been administered annually for many years (and after 2013, please, correct me if I am wrong), the authors could have investigated the longitudinal association between self-reported eyesight at baseline (in 2013) and mental wellbeing at follow-up, in a population who did not have mental ill-health at baseline.

It is true that HSE has been performed annually for many years, but the same participants are not included in every survey – a new sample is recruited each year. This allows for trend analyses, but not for longitudinal assessments of change within an individual (sadly!). This has been clarified in the discussion.

The 2013 survey was the most recent to include questions on eyesight, as this is one of the special topics which are only included in one year's version of the survey. This has been clarified in the discussion.

My second comment is that the authors could have presented age-stratified analyses (for example, in younger adults, middle-aged adults and older adults, respectively), while still adjusting for age. I would suggest age-stratified analyses in view of the wide age range of their study population

(16 to 104 years) and because the prevalence and risk factor for poorer wellbeing significantly vary across age categories.

We do not find a significant effect of age on wellbeing and did not have an a priori reason for performing an age-stratified analysis. However, we agree this is an interesting question so have re-run our models by age group (adolescents/emerging adults (age 16-29), adults (30-64) and older adults (65+)). The findings from these sensitivity analyses indicate that the findings hold across all age groups tested. Consequently, we include these analyses as a supplementary table only, indicating only in the main body of the text that they have been undertaken, (in the method and results sections and do not substantively change the findings.

Supplementary table 1. Regression analyses showing the regression of wellbeing on self-reported vision, mental ill-health, eye disease, demographic and socioeconomic variables. Values in bold indicate significant predictors at p<0.05. Reference levels for categorical predictors were (in brackets) as follows: self-reported vision (poor), mental ill-health (yes), eye disease (yes), gender (male), ethnicity (white British), socioeconomic status (routine or manual).

		**Adolescent and emerging adult (16-29 years, n=1252)**		**Adult (30-64 years, n=4577)**		**Older adult (≥65 years, n=1876)**	
**Predictor**	**Level**	Coefficient (95% CI)	*p* value	Coefficient (95% CI)	*p* value	Coefficient (95% CI)	*p* value
Self-reported vision	Excellent	**1.67 (0.65, 2.69)**	**<0.0001**	**6.78 (4.52, 9.04)**	**<0.0001**	**8.91 (6.30, 11.5)**	**<0.0001**
	Very good	**3.85 (0.09, 7.61)**	**<0.0001**	**5.34 (3.08, 7.60)**	**<0.0001**	**6.03 (3.50, 8.55)**	**<0.0001**
	Good	1.09 (-2.73, 4.92)	0.57	**3.94 (1.68, 6.21)**	**<0.0001**	**4.56 (2.05, 7.08)**	**<0.0001**
	Fair	-1.57 (-5.93, 2.80)	0.48	-0.26 (-2.68, 2.16)	0.83	1.42 (-1.29, 4.13)	0.30
Mental ill-health	No	**8.92 (7.27, 10.6)**	**<0.0001**	**11.5 (10.6, 12.5)**	**<0.0001**	**7.77 (5.32, 10.2)**	**<0.0001**
Eye disease	No	2.09 (-0.40, 4.58)	0.10	**1.68 (0.76, 2.61)**	**<0.001**	**1.76 (1.00, 2.53)**	**<0.0001**
Sex	Female	0.77 (-0.15, 1.68)	0.10	-0.04 (-0.54, 0.46)	0.88	0.71 (-0.05, 1.48)	0.07

Age		0.055 (-0.55, 0.55)	0.31	-0.02 (-0.046, 0.006)	0.08	**-0.156 (-0.21, -0.1)**	**<0.0001**
Ethnicity	Other	**1.65 (0.57, 2.73)**	**<0.01**	**1.39 (0.74, 2.03)**	**<0.0001**	1.11 (-0.50, 2.71)	0.18
Socioeconomic status	Higher managerial/ professional	**2.39 (1.33, 3.46)**	**<0.0001**	**3.44 (2.88, 4.00)**	**<0.0001**	**2.02 (1.13, 2.90)**	**<0.0001**
	Intermediate	0.74 (-0.48, 1.97)	0.23	**1.29 (0.61, 1.97)**	**<0.001**	0.97 (-0.01, 1.95)	0.05
	Other	-0.49 (-2.31, 1.32)	0.59	0.69 (-1.15, 2.54)	0.46	-0.49 (-3.47, 2.48)	0.75

I have a few other comments.

Title. Please, consider adding the word "cross-sectional" in the title. For example, "the cross-sectional association".

Changed as suggested, to:

> **The association between self-reported vision and mental wellbeing: A cross-sectional secondary analysis of Health Survey for England data**

Abstract. Please, consider reporting the age range and median age or mean age of the participants. Please, also consider reporting the proportion of women in your sample.

Thank you for this suggestion. We have added:

> **(56% women; median age 49 years, range 16-104 years)**

Strengths and weaknesses, page 4, lines 61-62. I would avoid the word "reduced" and change it with "poorer" or "lower"; in my view, the word "reduced" implies a change over time from a baseline. This study is only cross-sectional.

This sentence has been removed (see editor's first comment above).

Methods, page 9. Please, mention whether the Health Survey for England may be representative of the community-dwelling population in England. Please, provide appropriate references.

HSE is a representative sample of the population of England – this has been clarified with the phrase:

> **HSE includes a large number of participants, representative of the adult population of England**

(the Mindell reference at the end of this sentence supports this claim).

Methods, page 10, lines 169-170. What happened in case the participants had more than six conditions?

The interviewer guide specifies that only 6 coded conditions can be included, but each code is broad (for example, if someone reports depression and anxiety as two conditions, that would be included as one coded condition ('mental health'). The guide does not specify what happens if more than six groups of conditions are noted. Only 102 of the 7726 participants with responses to this question had six codes included, so the effect of these missing data is likely to be very small.

Methods, page 12. Please, consider mentioning whether the Health Survey for England received Ethics approval from an institutional review board. Please, report the name of the institutional review board and the date of approval. I understand that no further Ethics approval was required for secondary data

analyses, but you should report the original Ethics approval. Please, also mention whether written informed consent was received from all participants. Please, mention whether and how capacity to consent to participation in this study was assessed and whether potential participants lacking capacity were excluded from this study.
The original ethics approval has been recorded, as has the consent method used, which was (surprisingly) not written consent.

Findings, page 13. Please, consider a flow chart to show how the final analytical sample was reached.
This has been included, as figure 1.

Findings. You should provide a “Table 1 Characteristics of study population”; you could consider presenting the characteristics in the five vision categories. In other words, the headings of your columns will be “excellent”, “very good”, “good”, “fair” and “poor” self-reported vision. In the rows, you will report the proportion of women, the mean age, the proportion of the ethnicities, those of socio-economic status. You may also test for differences in each of these characteristics across the categories of self-reported vision (for example, using chi-square test for categorical variables).
This is an excellent suggestion, thank you. We have done exactly this.

Findings, page 13. What kind of test is F, please? It may be unclear to the reader.

This was an exact F-test, constructed using the method of least squares. This has been added to the statistical methods and results.

Discussion, page 17, lines 27-32. I am not sure whether the references correspond to these sentences. In the sentences, you mention three studies, but you only report two references, one of which refers to the English Longitudinal Study on Ageing rather than the Irish Longitudinal Study on Ageing.

Sorry, this was not clear. The two citations refer to four studies: the Whillans and Nazroo 2014 citation refers to the Irish Longitudinal Study on Ageing. The other paper, by Zimdars et al, includes analyses of the English Longitudinal Survey of Ageing, the MRC Assessment and Management of Older People in the Community Trial and the Health and Retirement Survey). To add to the confusion, there was a typo in the submitted paper – 'MEC' should read 'MRC' – this has been corrected.

Discussion, page 17, lines 8-19. Please, consider rephrasing this part by avoiding the use of the acronyms H1 and H2. For example, you could write "in a population of community-dwelling adults, we observed a cross-sectional association between poorer self-reported vision and poorer mental wellbeing".

We agree and have removed these abbreviations (see also response to reviewer 2, comment 2)

Discussion, page 18, lines 40-43. The statement "interventions to improve

vision are likely to improve wellbeing" is not supported by your observational cross-sectional study. You may rephrase it (for example, "further research" or "further interventional studies should explore whether interventions to improve vision are likely to improve wellbeing", or similar). You could also consider referencing previous interventional studies supporting this statement.

Yes, on re-reading we agree that this statement is too strong! We have removed this sentence but kept the recommendation for future research as suggested.

Discussion, page 18, lines 48-56. You should not present new results in the discussion. You should present them in the results section.

Sorry, these have now been moved to the results section.

Discussion, page 19, line 56. Please, consider changing "reduced wellbeing" with "poorer wellbeing" for the reason I explained above.

This has been changed as requested.

Discussion, page 19 and reference 30. I would reference a study on the provision of mental health support in physical health services for adult patients, rather than paediatric patients, given that your study focused on adults aged 16 to 104 years.

We have added an adult reference here (Wroe, 2015).

VERSION 2 - REVIEW

Reviewer 3

Name OGLIARI, GIULIA

Affiliation Nottingham University Hospitals NHS Trust, Health Care for Older People (HCOP)

Date 09-Jul-2025

COI

Thank you very much for revising your manuscript. I am happy with your revision.
